# *Aedes aegypti* var. *queenslandensis*: the pale phenotype of *Aedes aegypti* L. has landed in Amapá

**DOI:** 10.1590/0037-8682-0059-2025

**Published:** 2025-10-03

**Authors:** José Ferreira Saraiva, Dayse Swelen da Silva Ferreira, Felipe Greiner Amoras, Brenda Landrine Vilhena Nunes, Maria Luiza Cechim de Seixas Duarte, Fred Júlio Costa Monteiro, Ahana Maitra, Allan Kardec Ribeiro Galardo

**Affiliations:** 1 Instituto de Pesquisas Científicas e Tecnológicas do Estado do Amapá (IEPA), Laboratório de Entomologia Médica (LABENMED), Macapá, AP, Brasil.; 2 Instituto Nacional de Pesquisas da Amazônia (INPA), Programa de Pós-graduação em Ecologia, Manaus, AM, Brasil.; 3 Secretária Municipal de Vigilância em Saúde - SMVS, Prefeitura Municipal de Macapá, Macapá, AP, Brasil.; 4 Superintendência de Vigilância em Saúde do Amapá, Laboratório de Saúde Pública do Amapá-LACEN/AP, Macapá, AP, Brasil.; 5 University of Bari, Department of Biosciences, Biotechnologies and Environment, Bari, Italy.

**Keywords:** *Aedes* surveillance, Phenotypic diversity in mosquitoes, Invasive mosquito species

## Abstract

**Background:**

*Aedes aegypti* var. *queenslandensis*, a pale form of *Aedes aegypti*, is widespread in Asia and adapted to urbanized and warm environments.

**Methods:**

Two BG-Sentinel traps with BG-Lure were deployed in an urban forest fragment between December 19 and 24, 2024, to monitor mosquito vectors.

**Results:**

A total of 191 specimens of *Ae. aegypti* var. *queenslandensis* were collected, marking the first record of this variety in Macapá, Amapá.

**Conclusions:**

This finding suggests that the population was introduced via maritime transport and highlights the need to strengthen entomological surveillance and assess potential insecticide resistance in vector control efforts in Macapá.

*Aedes aegypti*(Linnaeus, 1762) is the primary vector of several medically important arboviruses worldwide, including dengue, Zika, chikungunya, and yellow fever^1^. These vector-borne diseases present serious public health challenges, particularly in tropical and subtropical regions^2^. The rapid expansion of the geographic distribution of*Ae. aegypti*, driven by urban growth and climate change, has further exacerbated the global burden of these infections^3^.

In Macapá, in the state of Amapá (Brazil), dengue cases have risen sharply in recent years, increasing from 620 cases in 2023 to 5,701 cases in 2024. This situation is worsened by the simultaneous circulation of three dengue virus serotypes (DENV1, DENV2, and DENV3), which increases the likelihood of severe manifestations of the disease, such as dengue hemorrhagic fever^4^. In this context, a comprehensive understanding of the genetic and phenotypic diversity of *Ae. aegypti* is essential to assess possible changes in vector fitness and resistance to insecticides commonly used in local control programs. The introduction of exotic phenotypes poses an additional risk, as these variants often exhibit greater adaptability to urban environments and elevated temperatures^5^.

The three main forms of *Aedes aegypti* display striking morphological differences: *Ae. aegypti aegypti* (dark brown), *Ae. aegypti formosus* (black), and *Ae. aegypti* var. *queenslandensis* (pale white)^6^ ^,^ ^7^. The brown-colored *Ae. aegypti aegypti*, the type form, is the most widely recognized subspecies and the primary urban vector of arboviruses, characterized by light scales restricted to the first abdominal tergite. By contrast, *Ae. aegypti formosus*, black in coloration, is a sylvatic subspecies found in African forests. Meanwhile, *Ae. aegypti* var. *queenslandensis*, although genetically indistinguishable from *Ae. aegypti aegypti* ^8^ and not considered a separate subspecies, can be distinguished by its pale white pattern or gradient of white scales covering the abdominal tergites, and it is typically associated with urban environments and warm climates^9^.

Although *Ae. aegypti* var. *queenslandensis* is described as a variety rather than a subspecies, its phenotype has been reported in urban areas of Australia, Indonesia, and Mediterranean regions, standing out for its remarkable adaptation to anthropogenic environments. In Brazil, its presence was recently documented in Taubaté, São Paulo^10^. Here, we report its occurrence in Amapá, further expanding its known distribution (Figure 1). 

**FIGURE 1:**
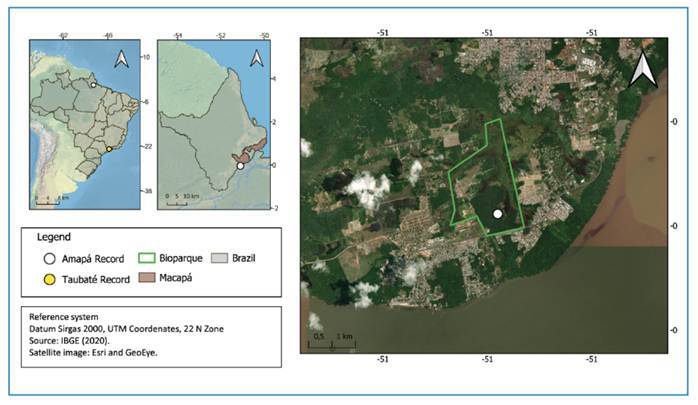
Geographic location of the *Aedes aegypti* var. *queenslandensis* collection site in Macapá, Amapá, Brazil. The maps show: (i) the national context with collection records in Macapá (**white circle**) and Taubaté (**yellow circle**); (ii) the regional location of Macapá municipality (**in brown**); and (iii) a high-resolution satellite image of the Bioparque (**green polygon**), where BG-Sentinel traps were deployed. The white circle marks the exact collection site within the park, located near forest-edge and peri-urban transition zones.

In the state of Amapá, *Ae. aegypti* var. *queenslandensis* was recorded during an entomological monitoring survey at one of the anthropized edges of the Amazon Biopark (0° 2'27.00" S; 051° 5'46.33" W), located along the Josmar Chaves Pinto Highway in Jardim Marco Zero, Macapá. A total of 191 female *Ae. aegypti* specimens displaying distinct phenotypic variations from the type form were collected (Figure 2). Sampling was carried out using two BG-Sentinel (BGS) traps baited with BG-Lure (BGL) [Trap 01: 0° 2'17.38" S; 51° 5'39.32" W; Trap 02: 0° 2'18.78" S; 051° 5'37.28" W]. The traps were inspected daily at 10:00 a.m. for five consecutive days (December 19-24, 2024). Among the collected samples, five specimens exhibited completely white abdomens, corresponding to *Ae. aegypti* var. *queenslandensis* form F. The remaining 186 specimens displayed varying degrees of abdominal white scaling, classified on a gradient from A-E according to McClelland^7^, indicating intermediate phenotypes (Table 1). All voucher specimens were deposited in the entomological collection of the Instituto de Pesquisas Científicas e Tecnológicas do Amapá (IEPA) under voucher numbers #12127 - #12134.

**FIGURE 2:**
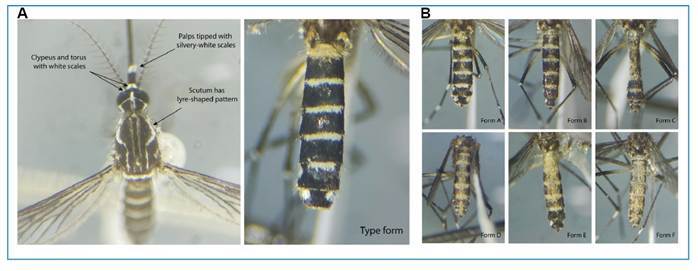
Abdominal scale pattern variation in *Aedes aegypti* var. *queenslandensis* females collected in December 2024 in Macapá, Amapá, Brazil, using BG-Sentinel traps with BG-Lure. The images illustrate distinct abdominal morphotypes based on the distribution of pale scales on the first tergites, corresponding to the forms described by McClelland (1974). The “*queenslandensis*” form shows extensive pale scaling extending laterally and posteriorly, whereas darker forms exhibit restricted or fragmented pale scale patches. These morphological variations reflect intraspecific diversity within *Ae. aegypti* and may aid in field identification.

**TABLE 1:** Number of specimens collected by species and phenotype in BGS traps with BGL at the edge of an anthropized area in Macapá, Amapá, during the study period from December 19 to 24, 2024.

Species/Phenotypes	N	x̄	%
*Aedes albopictus*	142	23,7	11,3
*Ae. aegypti* type form	919	153,2	73,4
*Ae. a.* var. *queenslandensis* form A	75	12,5	6,0
*Ae. a.* var. *queenslandensis* form B	83	13,8	6,6
*Ae. a.* var. *queenslandensis* form C	21	3,5	1,7
*Ae. a.* var. *queenslandensis* form D	6	1	0,5
*Ae. a.* var. *queenslandensis* form E	1	0,2	0,1
*Ae. a.* var. *queenslandensis* form F	5	0,8	0,4

The phenotype of *Ae. aegypti* var. *queenslandensis* is controlled by quantitative inheritance, with at least three QTLs (Quantitative Trait Loci) mapped to chromosomes 1, 2, and 3, the QTL on chromosome 2 being the primary determinant of the light scale pattern^9^. The phenotypic gradient observed in the Macapá population may result from genetic recombination between distant phenotypic forms or environmental influences on scale pattern expression^5^. This variability suggests the coexistence of different genotypes in the region and the potential intercrossing of *Ae. aegypti* var. *queenslandensis* with the local-type form.

Similar patterns have been reported in previous population genetics studies of *Ae. aegypti* across different Brazilian regions, where the greatest genetic differences were observed among populations from Macapá, Belém, and Santarém compared to other areas of the country^11^ ^,^ ^12^. Belém, a major port city located approximately 100 km upriver from the Atlantic Ocean, serves as a key gateway to the Amazon River and facilitates the introduction of new genetic groups of *Ae. aegypti.* Similarly, Macapá lies along the northern channel of the Amazon River, close to the Atlantic Ocean, and shares borders with Suriname and French Guiana. These cities and their surrounding areas may therefore represent important entry points for novel genetic variants of this vector. Maitra et al.^13^ also observed the highest number of private alleles in the *Ae. aegypti* population from Macapá, suggesting the existence of a distinct genetic subgroup separate from other Brazilian populations.

The presence of *Ae. aegypti* var. *queenslandensis* in Macapá may indicate an introduction through the port of Santana (9.5 km from the collection site), a major entry point for large international vessels. This hypothesis is further supported by previous records of another invasive species, *Aedes albopictus* (Skuse, 1894), which was first detected near the port in 2019^14^, and is now widespread in Macapá^15^. Historical data show that *Ae. aegypti* var. *queenslandensis* occurs in Australia, Southeast Asia, the Mediterranean, and São Paulo, Brazil, suggesting that its introduction into Macapá may have originated from one of these regions^3^.

Given this scenario, it is essential to strengthen entomological surveillance at the port of Santana and other ports in the state, given their strategic role as potential entry points for invasive species. This measure is critical not only for monitoring the spread of *Ae. aegypti* var. *queenslandensis* but also for mitigating its potential impact on arbovirus transmission in Amapá, considering the possible introduction of new populations and phenotypes better adapted to urban environments. Implementing robust preventive strategies will enable a more effective response to the challenges posed by the genetic diversity of *Aedes aegypti* ^2^, particularly with regard to its vectorial capacity and resistance to insecticides used in local control programs. Future research should prioritize genetic characterization and expanded surveillance in key ports to track further introductions and strengthen public health interventions.

## Data Availability

Data-in-article.
